# Intrastromal autologous blood injection for treating corneal hydrops

**DOI:** 10.5935/0004-2749.2025-0120

**Published:** 2025-09-10

**Authors:** Lucas Baldissera Tochetto, Flavio Eduardo Hirai, Ítalo Pena de Oliveira, Klaus Tyrrasch, Tais Hitomi Wakamatsu, José Álvaro Pereira Gomes

**Affiliations:** 1 Department of Ophthalmology and Visual Science, Escola Paulista de Medicina, Universidade Federal de São Paulo, São Paulo, SP, Brazil

**Keywords:** Corneal edema, Corneal diseases, Edema, Visual acuity, keratoconus

## Abstract

**Purpose:**

To describe the technique and outcomes of intrastromal autologous blood injection in patients with severe corneal hydrops.

**Methods:**

Nineteen patients with corneal hydrops underwent intrastromal autologous blood injection. Postoperative assessments included best-corrected visual acuity and time to resolution of corneal edema.

**Results:**

Corneal edema resolved within 1 week in 5 patients, within 1 month in 11, and within 3 months in 3. The mean duration of edema persistence was 37.94 ± 33.05 days (range, 6–124). Corneal thickness decreased from 2.06 ± 0.71-mm preoperatively to 1.34 ± 0.65-mm at day 7, 0.85 ± 0.56-mm at day 30, and 0.57 ± 0.13-mm at day 90 (p<0.001). Descemet’s membrane (DM) detachment decreased from 1.01 ± 0.75-mm to 0.44 ± 0.57-mm, 0.24 ± 0.36-mm, and 0.08 ± 0.11-mm on postoperative days 7, 30, and 90, respectively (p<0.001). DM break size decreased from 1.12 ± 1.19-mm to 0.62 ± 0.84-mm at 3 months (p<0.005). Three patients developed hematocornea; no other major complications were observed. At 3 months, mean best-corrected visual acuity improved from 2.37 ± 0.66 to 0.41 ± 0.17 logMAR with hard contact lenses (p<0.001).

**Conclusions:**

Intrastromal autolo gous blood injection is an effective treatment for severe corneal hydrops, promoting faster edema resolution and visual improvement with minimal complications.

## INTRODUCTION

Acute corneal hydrops is a complication of several corneal ectatic disorders, including keratoconus, pellucid marginal degeneration, keratoglobus, and Terrien’s marginal degeneration^(1^,^2)^. It results from rupture and separation of Descemet’s membrane (DM) and the endothelium, allowing aqueous humor to enter the stroma and cause focal stromal edema, intrastromal clefts, and fluid-filled cysts^(3)^.

The natural course involves spontaneous resolution of edema within 5–36 weeks^(1^,^4^,^5)^. Severe cases with extensive DM lesions may lead to persistent edema and complications such as permanent opacities, neovascularization, rupture of subepithelial cysts with pain, corneal perforation, and infectious keratitis^(5^-^7)^. To accelerate edema resolution, improve best-corrected visual acuity (BCVA), and optimize conditions for future keratoplasty, several invasive interventions have been described^(1^,^8^,^9)^. These include intracameral air or gas (SF6 or C3F8) injection, pre-DM sutures, thermokeratoplasty, compression sutures, and posterior lamellar keratoplasty^(1^,^8^,^10^-^13)^. However, most of these approaches either damage corneal tissue or require intraocular access, carrying risks such as pupillary block, gas-induced intraocular pressure elevation^(1^,^3^,^14)^, Urrets–Zavalia syndrome^(15)^, and cataract formation^(16^,^17)^.

The intrastromal injection of autologous blood offers a means of coating the posterior surface of the defective cornea, creating a mechanical barrier against aqueous humor and promoting regeneration through growth factors and bioactive proteins present in the blood^(18)^. Blood-derived therapies have shown efficacy and safety in ocular surface disease^(19^,^20)^, postfiltration bleb leaks^(21)^, and severe chronic ocular hypotension following glaucoma surgery^(22)^. However, reports on the use of intrastromal autologous blood for corneal hydrops are limited to a few case studies^(18)^.

This study aimed to evaluate the efficacy and safety of intrastromal autologous blood injection for the management of severe corneal hydrops.

## METHODS

### Study participants

Nineteen consecutive patients with acute corneal hydrops underwent intrastromal autologous blood injection at the outpatient clinic of the *Universidade Federal de São Paulo*, Brazil, between April 2021 and November 2021. Inclusion criteria were disease duration <2 months and corneal edema grade 2 or 3. Exclusion criteria included ocular surgery within the past 6 months, infectious keratitis, corneal perforation or athalamia, and clinical improvement with conservative treatment.

### Ethical considerations

The study adhered to the principles of the Declaration of Helsinki (2013 revision) and was approved by the institutional review board (0915P/2021). Written informed consent was obtained from all patients or, when applicable, their parents/guardians.

### Hydrops definition and evaluation

Acute corneal hydrops, caused by DM rupture and stromal edema, presents as sudden vision loss in the affected eye with underlying corneal ectasia. The condition is graded according to the extent of corneal edema: grade 1, edema ≤3-mm in diameter; grade 2, 3–5-mm; and grade 3, >5-mm^(23)^.

Risk factors and associated symptoms were recorded. All patients underwent comprehensive ophthalmic examination, including BCVA, slit-lamp photography, and tonometry, before the procedure and at postoperative day (POD) 1, week 1, month 1, and month 3. Intraocular pressure was measured using an ICare rebound tonometer (ICare Finland, Oy, Finland). Anterior segment optical coherence tomography (AS-OCT; Visante OCT, Carl Zeiss Meditec, Dublin, CA, USA) was performed preoperatively and at 1 week, 1 month, and 3 months postoperatively. Corneal parameters were measured with calipers (mm): (1) maximum corneal thickness (distance between anterior and posterior surfaces in the area of greatest edema); (2) depth of DM detachment (distance between posterior corneal surface and detached DM); and (3) size of DM break (distance between broken DM ends). Resolution was defined as complete disappearance of epithelial cysts and stromal edema with subsequent stromal scarring, as confirmed by slit-lamp biomicroscopy and AS-OCT.

### Surgical procedure

**Figure 1:**
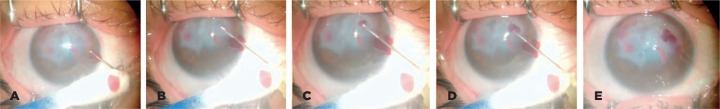
Surgical management of acute corneal hydrops with intrastromal autologous blood injection. (A) Cannula introduced into the clear cornea; (B) advanced to the cystic region; (C, D) cyst filled with blood to replace intracystic aqueous humor; (E) final corneal appearance postprocedure.

All procedures in the operating room under topical anesthesia (1% tetracaine hydrochloride/0.1% phenylephrine hydrochloride) and sterile conditions following periocular preparation with 10% povidone–iodine. A 3-ml sample of peripheral venous blood was obtained from the upper limb. Approximately 100 µl of autologous blood was injected into the corneal stroma using a 30-gauge, 8-mm cannula manually bent to ~45° to facilitate controlled injection under the surgical microscope. The needle bevel was directed toward the corneal stroma adjacent to the aqueous humor-filled stromal cyst, allowing the injected blood to occupy the intracystic space. Entry was made through an area of the cornea free of cysts, with the cannula advanced into the affected region (Figure 1). Postoperatively, patients received a topical antibiotic–steroid combination for 7 days, followed by prednisolone 0.1% four times daily for 90 days.

### Statistical analysis

Continuous avariables were expressed as mean ± standard deviation, and categorical variables as frequency (%). Pre- and postoperative comparisons were performed using the Wilcoxon signed-rank test. Longitudinal changes were assessed with regression models based on the generalized estimating equation method. A p-value <0.05 was considered statistically significant. All analyses were conducted using Stata version 17 (StataCorp, College Station, Texas, USA).

## RESULTS

The study included 19 patients (12 men [63%], 7 women [37%]) with a mean age of 21.8 ± 10.6 years. Eighteen patients had keratoconus and one had keratoglobus. The mean interval between hydrops onset and treatment was 35.8 ± 32.6 days (range, 6–153). Cohort characteristics are summarized in table 1.

**Table 1:** Demographics and clinical characteristics of the study population

Age (years)	21.89 ± 10.66
Sex	
Male	12 (63.16%)
Female	7 (36.8%)
Affected eye	
Right	8 (42.11%)
Left	11 (57.89%)
Ethnicity	
White	3 (15.79%)
Black	16 (84.21%)
Risk factors	
Diagnosis of keratoconus before age 15	9 (47.37%)
Eye rubbing	18 (94.74%)
Vernal keratoconjunctivitis	4 (21.05%)
Atopic keratoconjunctivitis	1 (5.26%)
Seasonal or perennial allergic conjunctivitis	13 (68.42%)
Asthma	4 (21.05%)
Hydrops grade	
Grade 1	0 (0%)
Grade 2	5 (26.32%)
Grade 3	14 (73.68%)
Localization	
Central	8 (42.11%)
Peripheral	11 (57.89%)

At the time of the procedure, blood leakage into the anterior chamber (AC) was observed in 11 patients (57.9%). On postoperative day (POD) 1, leakage persisted in five patients (26.3%) but resolve spontaneously within a few days. Intraocular pressure remained within the normal range throughout follow-up. Two patients had positive Seidel test results on POD 1; both were controlled with compressive dressings, applied for 1 day in one patient and 3 days in the other.

**Figure 2:**
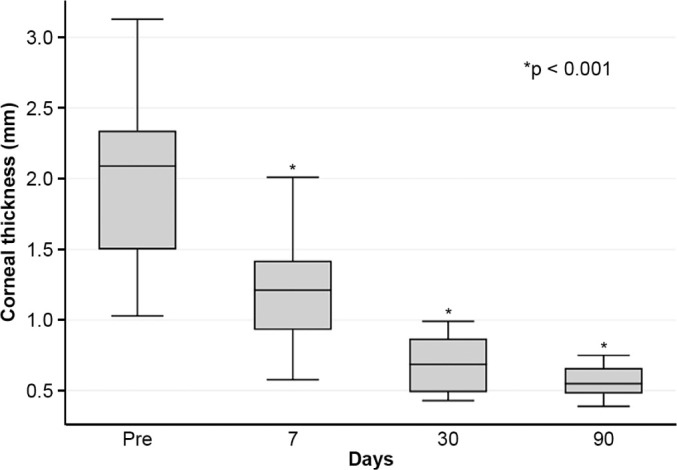
Changes in corneal thickness in patients with acute corneal hydrops before and after autologous blood injection.

**Figure 3:**
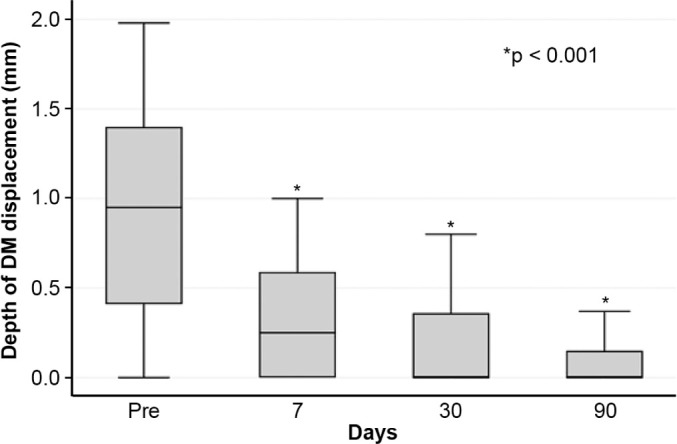
Descemet membrane detachment in patients with acute corneal hydrops before and after autologous blood injection.

**Figure 4:**
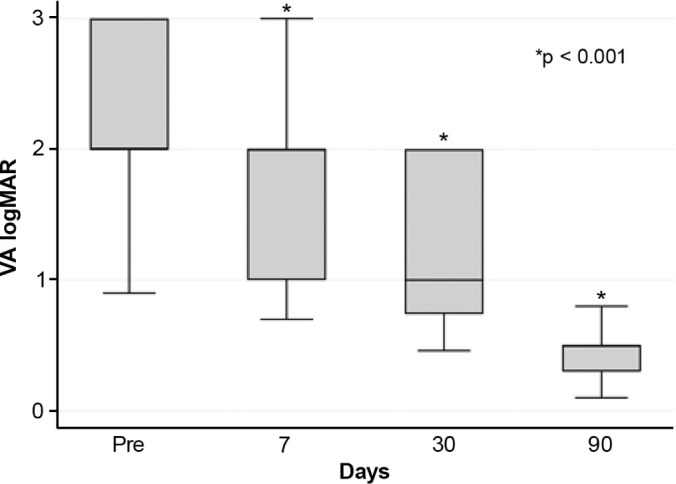
Descemet membrane rupture sizes in patients with acute corneal hydrops before and after autologous blood injection.

The mean corneal thickness progressively decreased from 2.06 ± 0.71-mm at baseline (POD 0) to 1.34 ± 0.65-mm on POD 7, 0.85 ± 0.56-mm on POD 30, and 0.57 ± 0.13-mm on POD 90 (p<0.001) (Figure 2). A similar trend was observed for DM detachment, which decreased from 1.01 ± 0.75-mm at baseline to 0.44 ± 0.57-mm on POD 1, 0.24 ± 0.36-mm on POD 7, 0.24 ± 0.36-mm on POD 30, and 0.08 ± 0.11-mm on POD 90 (p<0.001) (Figure 3). The mean size of the DM tear decreased from 1.12 ± 1.19-mm to 0.62 ± 0.84-mm at 3 months (p<0.005) (Figure 4). At the end of follow-up, eight patients (42.1%) still exhibited residual rupture and detachment of the DM. The mean duration of corneal edema persistence was 37.94 ± 33.05 days (range, 6–124). Edema resolved within 7 days in 5 patients (26.3%), within 1 month in 11 patients (57.9%), and within 3 months in 3 patients (15.8%). By POD 30, 84.2% of patients showed resolution, and by POD 90, all patients (100%) had complete resolution.

**Figure 5:**
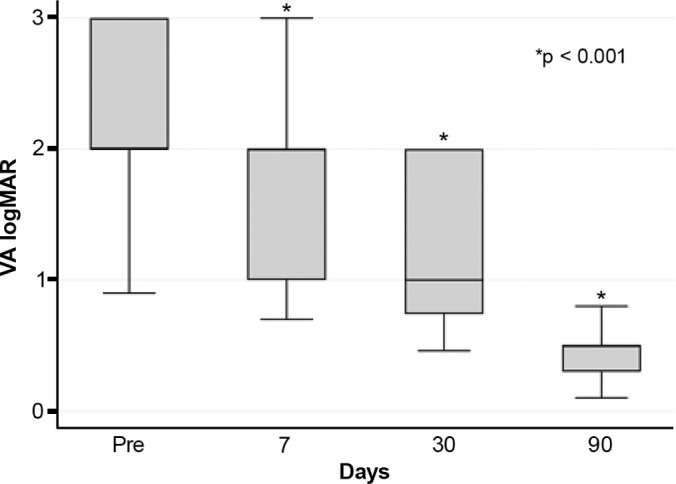
Best-corrected visual acuity in patients with acute corneal hydrops before and after autologous blood injection.

Mean BCVA improved significantly from 2.37 ± 0.66 logMAR preoperatively to 0.41 ± 0.17 logMAR at 3 months postoperatively with hard contact lens correction (p<0.001) (Figure 5).

No ocular hypertension was observed. At treatment initiation, three patients had corneal neovascularization, and one additional case developed on POD 7.

**Figure 6:**
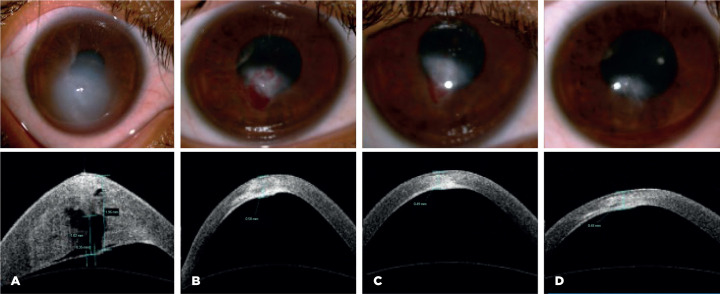
Clinical and anterior segment OCT images of keratoconus with corneal hydrops and intrastromal cleft. (A) At presentation; (B) 1 week after autologous blood injection; (C) 1 month post-injection; (D) 3 months postinjection.

Intrastromal blood resolved within the first month in five patients and by 3 months in another five (Figure 6). In six patients, the blood persisted in the stromal region throughout the study period. The remaining three patients developed hematocornea–one within 7 days and two within 1 month postoperatively.

## DISCUSSION

Acute corneal hydrops is a complication of corneal ectatic disorders, occurring in 2.6%–7.5% of patients with keratoconus^(4^,^24^,^25)^ and in 11%–14.7% of those with keratoglobus^(1)^. It results from rupture of DM and the endothelium, allowing aqueous humor to enter the corneal stroma and form large fluid-filled stromal clefts or cysts^(26)^. Endothelial migration from adjacent intact areas over the exposed stroma contributes to resolution by partially reestablishing a barrier, limiting fluid ingress and reducing stromal edema^(4^,^26)^. Current treatments focus on accelerating edema clearance and preventing secondary complications, such as infectious keratitis^(27)^, perforation^(28)^, and corneal neovascularization^(29)^.

Several studies have reported successful treatment of corneal hydrops with intracameral injection of air, C3F8, or SF6. These gases act as mechanical barriers within the AC, limiting aqueous flow through the DM rupture and facilitating DM reattachment^(8^,^14)^. Miyata et al.^(14)^ demonstrated faster resolution of corneal edema with intracameral air injection–20.1 ± 9.0 days compared with 64.7 ± 34.6 days in the control group^(14)^. However, repeated injections are often required. Basu et al. reported mean resolution times of 78.7 (± 53.2) days in eyes treated with C3F8 versus 117.9 (± 68.2) days in controls (p<0.0001). Despite this, no significant benefit was seen in cases of inferior peripheral pellucid marginal degeneration, keratoglobus, or when large DM tears were present. In such cases, gas tamponade may be insufficient to seal the rupture or achieve stable DM reattachment^(1)^.

In this study, we evaluated 19 patients with acute corneal hydrops treated with intrastromal autologous blood injection. The main findings were significant reductions in corneal thickness and DM detachment, improvement in BCVA, and complete resolution of corneal edema within a maximum of 3 months. All 19 patients showed clinical improvement, with edema resolution occurring in an average of 37.94 (± 33.05) days. This outcome was superior to that reported by Basu et al. with intracameral C3F8 injection^(1)^. Although our results were not as rapid as those of Miyata et al. with intracameral air injection, it is important to highlight that most patients in their series required multiple air injections, whereas a single procedure was sufficient in our study^(14)^.

When comparing corneal edema measurements with a similar study using SF6 injection into the AC, our cohort showed a faster and more pronounced reduction in corneal thickness at 90 days (0.57-mm versus 0.649-mm)^(8)^. The combination of corneal compressive sutures with SF6 injection has been reported to further accelerate pachymetric recovery (0.66 mm after 7 days). However, this technique presents notable technical challenges, particularly the placement of sutures without direct visualization of the DM, in addition to the potential risk of complications^(30)^.

Blood-derived products contain multiple growth factors, nutrients, and proteins that promote tissue repair and regeneration. Consequently, their use in ocular surface disease management has increased in recent years^(19^,^20)^. A case report described successful treatment of severe corneal hydrops in a patient with Down’s syndrome using intracameral eye platelet-rich plasma (E-PRP)^(18)^. The E-PRP was prepared with sodium nitrate as an anticoagulant followed by centrifugation to concentrate platelets in the plasma fraction. In contrast, the present study favored autologous blood applied directly to the intrastromal cornea, as this approach avoids blood preparation or AC access, thereby simplifying treatment and lowering the risk of intraocular complications.

Complications have been reported with all procedures used to treat hydrops, and this risk must be carefully weighed before recommending an invasive intervention for a condition that can resolve spontaneously. Most studies, including the present one, reserved invasive treat ment for patients with grade 2 or 3 hydrops, in whom the risks of disease-related complications outweighed those of iatrogenic harm. Two controlled comparative studies of gas versus air injection demonstrated a 1.5- to 3.2-fold faster resolution of corneal edema. Another consideration is the optimal timing of intervention. Panda et al. reported better outcomes with gas injection performed within the first week of hydrops^(8)^, and other studies also initiated invasive treatment early in the disease course. In contrast, the present study delayed intervention for several weeks, allowing observation of corneal edema and symptoms to better define disease progression and stage.

Among the reported complications of invasive procedures for hydrops, pupillary block and secondary glaucoma are the most feared, as both can cause permanent visual loss. This risk is particularly associated with air or gas injection into the AC. Basu et al. reported that 10 of 62 patients (16.1%) developed increased intraocular pressure, including 7 cases of pupillary block that required pressure decompression^(1)^. In contrast, in the present study, intraoperative blood leakage into the AC occurred in 11 of 19 patients (57.9%), leading to small postoperative hyphema in 5 cases. These hyphemas were transient, resolving within a few days without intraocular pressure elevation or other complications. We also observed that the injected blood was reabsorbed over time without color changes in most cases. Nevertheless, three patients (15.8%) developed a localized, dense rust-colored opacity at the injection site; in two of these, visual acuity was ≤20/100 in the visual axis. Following hydrops resolution, the remaining 17 patients (89.5%) achieved BCVA of 20/60 or better with hard contact lenses.

AS-OCT is a valuable tool for detecting and diagnosing intrastromal clefts and for monitoring edema resolution through corneal thickness measurements^(26)^. Most published studies on hydrops treatment did not incorporate AS-OCT evaluation and may therefore have overlooked important aspects of the tissue repair mechanism. In our series, AS-OCT analysis revealed a rapid reduction in corneal thickness, as well as a decrease in the extent of DM rupture and detachment, within just a few days after surgery.

This study, however, had several limitations. It lacked a control group, and treatment success was assessed clinically by the disappearance of corneal edema rather than by objective parameters such as corneal thickness. The latter was not possible because pre-edema thickness values were unavailable; making it difficult to determine which postoperative measurements represented a true return to normal. Notably, eight patients continued to show DM rupture and detachment even after edema had resolved. The introduction of blood into stromal cysts likely blocked aqueous humor ingress and promote scarring, obviating the need to reposition the DM.

In conclusion, this study highlights the effectiveness and safety of autologous blood in managing corneal edema associated with hydrops. Larger prospective comparative trials are warranted to confirm these results and to define the broader applicability of this approach. Addressing the current study’s limitations will further clarify its therapeutic potential.

## Data Availability

The datasets generated and/or analyzed during the current study are already available.
